# Supplementary material for: Detecting heritable phenotypes without a model using fast permutation testing for heritability and set-tests
Source: Nat Commun. 2018 Nov 21;9:4919. doi: 10.1038/s41467-018-07276-w (PMC6249264; doi:10.1038/s41467-018-07276-w)
Supplement: Supplementary file 1 — Supplementary Information [file 41467_2018_7276_MOESM1_ESM.pdf]

Supplementary Information - Detecting heritable phenotypes without a model using fast permutation testing for heritability and set-tests

Regev Schweiger[1,*], Eyal Fisher[2], Omer Weissbrod[3], Elior Rahmani[1], Martina Müller-Nurasyid[4,5,6], Sonja Kunze[7,8], Christian Gieger[7,8], Melanie Waldenberger[6,7,8], Saharon Rosset[2] and Eran Halperin[9,10]

[1]Blavatnik School of Computer Science, Tel Aviv University, Tel Aviv, Israel
[2]School of Mathematical Sciences, Department of Statistics, Tel Aviv University, Tel Aviv, Israel
[3]Department of Epidemiology, Harvard T.H. Chan School of Public Health, Boston, MA, USA
[4]Institute of Genetic Epidemiology, Helmholtz Zentrum München - German Research Center for Environmental Health, Neuherberg, Germany
[5]Department of Medicine I, Ludwig-Maximilians-Universität, Munich, Germany
[6]DZHK (German Centre for Cardiovascular Research), partner site Munich Heart Alliance, Munich, Germany
[7]Institute of Epidemiology II, Helmholtz Zentrum München - German Research Center for Environmental Health, 85764 Neuherberg, Germany
[8]Research Unit of Molecular Epidemiology, Helmholtz Zentrum München - German Research Center for Environmental Health, 85764 Neuherberg, Germany
[9]Department of Computer Science, University of California, Los Angeles, CA, USA
[10]Department of Anesthesiology and Perioperative Medicine, University of California, Los Angeles, CA, USA
[*]Corresponding author: schweiger@post.tau.ac.il

Supplementary Figures

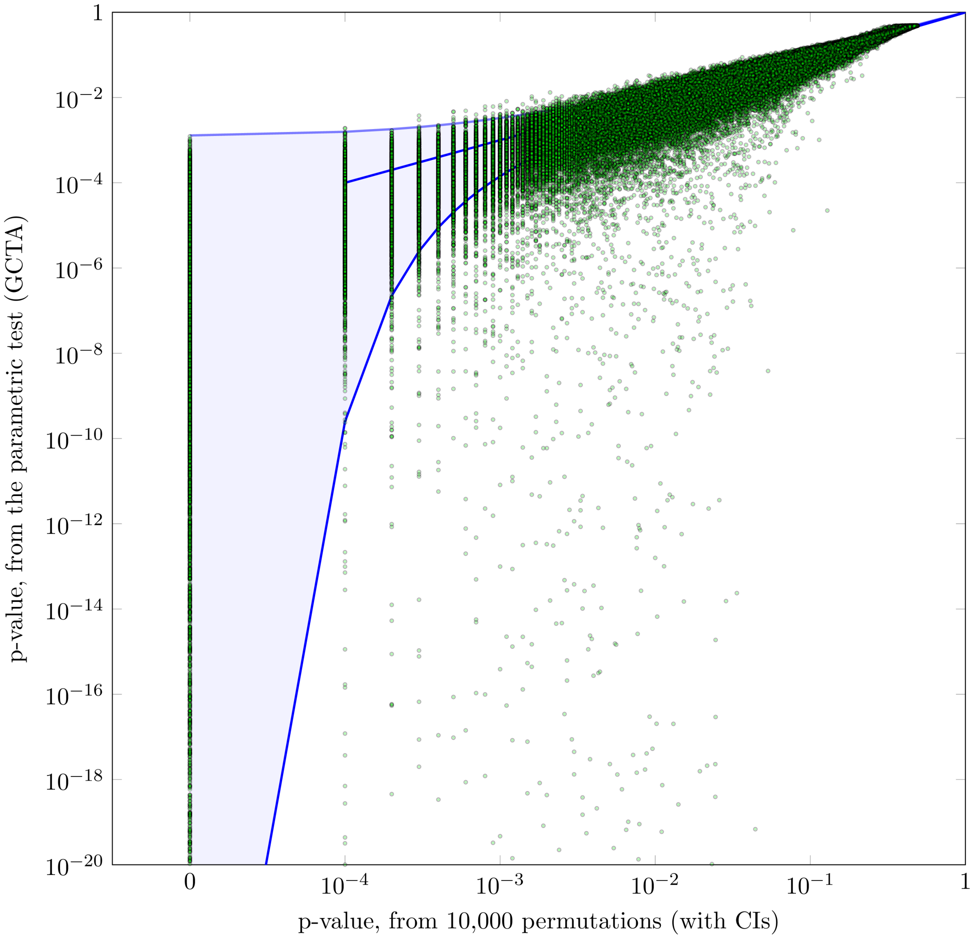

Supplementary Figure 1 Discrepancy in p-values in a methylation study. p-values from 10,000 permutations, compared to GCTA p-values assuming asymptotics (in log scale). Evaluated on 431,366 methylation sites on all autosomal chromosomes, from the KORA dataset, with 1,799 individuals. Sites with $\hat{h}^2 = 0$ (206,436 sites) or with a parametric $p < 10^{-20}$ (570 sites) omitted for clarity of presentation, showing a total of 224,360 sites, with 99.995% confidence intervals (CIs). Parametric p-values are often smaller than the exact p-values obtained by the permutation test, frequently by several orders of magnitude, resulting in many false positives.

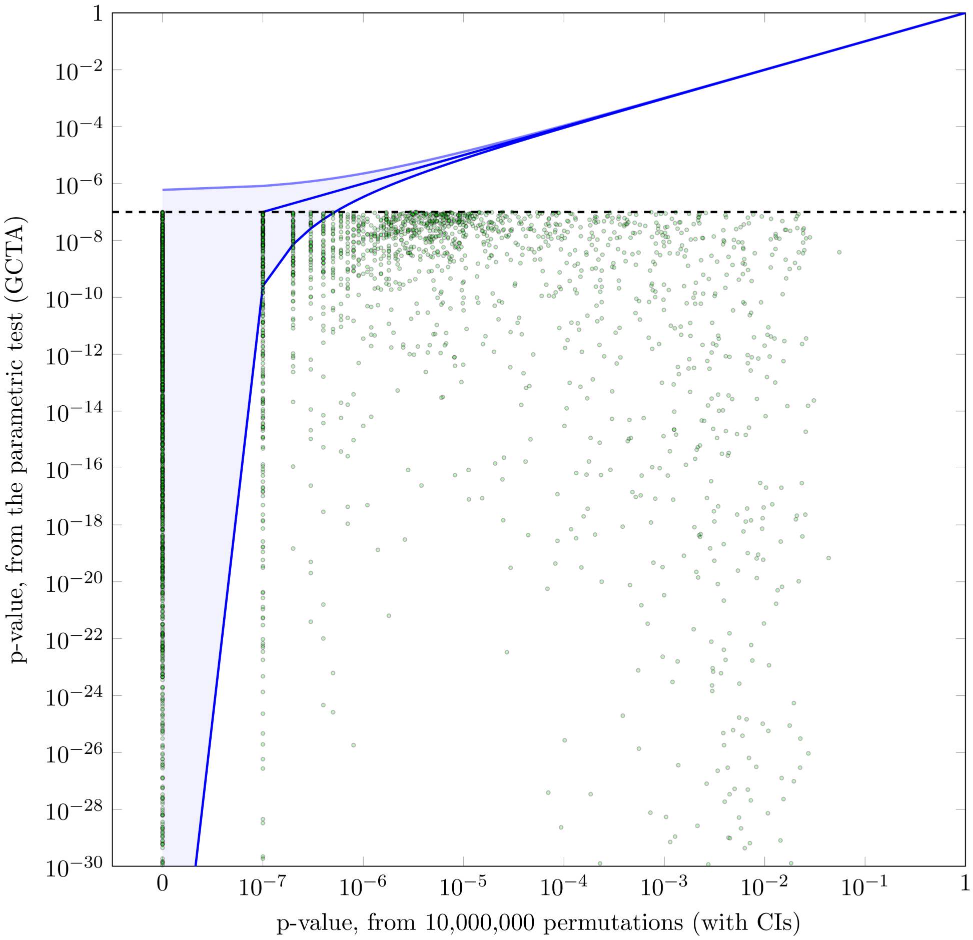

Supplementary Figure 2 Discrepancy in p-values. p-values from 10,000,000 permutations, compared to GCTA p-values assuming asymptotics (in log scale). Evaluated on 3,489 methylation sites for which the GCTA p-value is $p < 10^{-7}$, on all autosomal chromosomes, from the KORA [18] dataset. Sites with a parametric $p < 10^{-30}$ (143 sites) omitted for clarity of presentation, showing a total of 3,346 sites, with 99.5% CIs. Parametric p-values are still often smaller than the exact p-values obtained by the permutation test by several orders of magnitude.

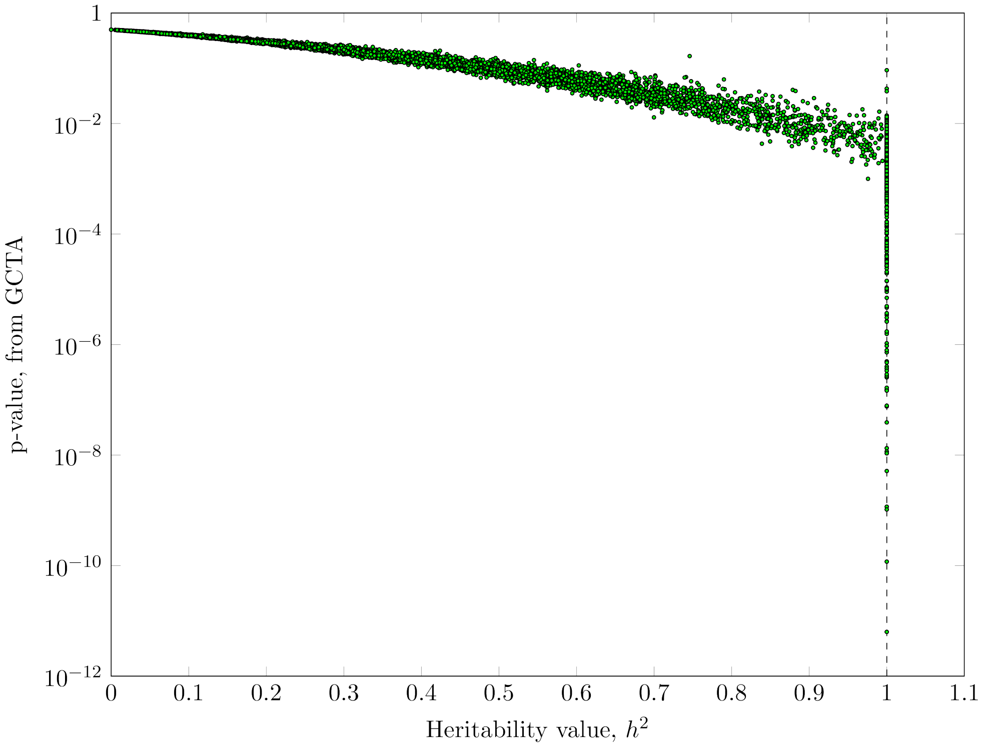

Supplementary Figure 3 GTEx heritability study. Heritability values of gene expression profiles of the GTEx study, compared with their respective parametric p-values. When $\hat{h}^2 = 1$ is estimated, p-values are uncalibrated, resulting in discrepancies.

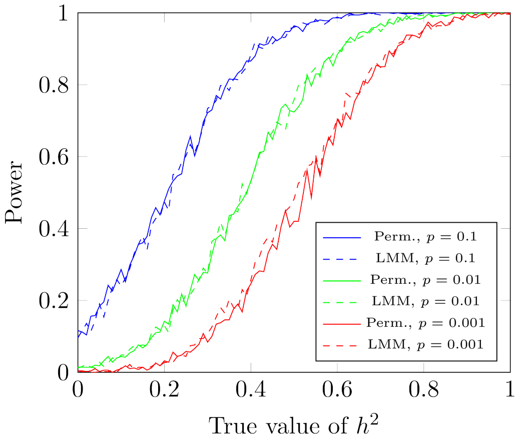

Supplementary Figure 4 Power study. The power of the permutation test (full lines) and the LMM parametric test (dashed lines) is shown for p-value thresholds of $p = 0.1, 0.01$ and 0.001. The power of the two tests is comparable.

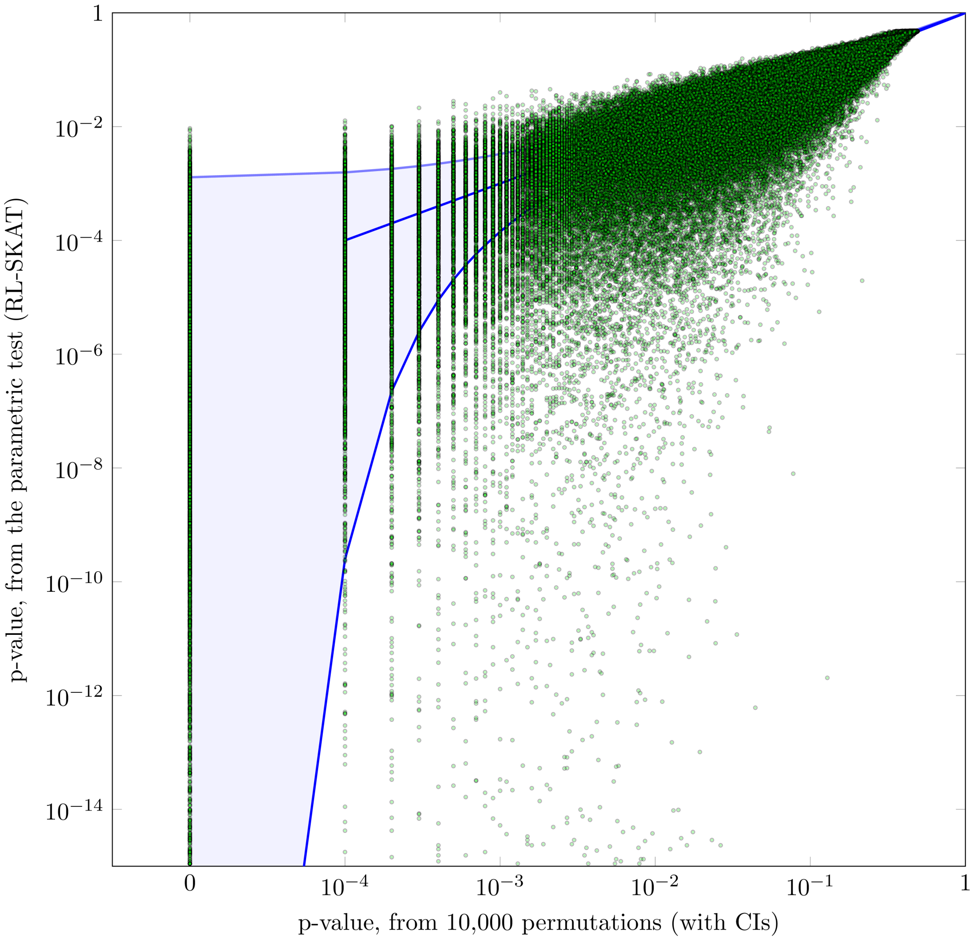

Supplementary Figure 5 Discrepancy in p-values. p-values from 10,000 permutations, compared to SKAT p-values, as implemented by RL-SKAT (in log scale). Evaluated on 431,366 methylation sites on all autosomal chromosomes, from the KORA dataset, with 1,799 individuals. Sites with $\hat{h}^2 = 0$ (206,436 sites) omitted for clarity of presentation, showing a total of 224,930 sites, with 99.995% CIs. Parametric p-values are often smaller than the exact p-values obtained by the permutation test, frequently by several orders of magnitude, resulting in many false positives.

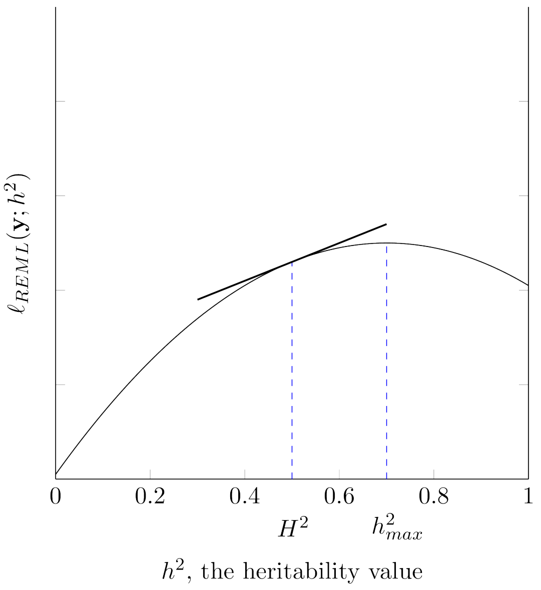

Supplementary Figure 6 Illustration of the derivative-based approach. The logarithm of the restricted likelihood function is shown as a function of the heritability value, h^2. The true REML estimate is shown by h^2_{max}, while the observed heritability estimate is shown by H^2. The derivative of the log-restricted-likelihood function at H^2 is shown as a linear slope, and it points to the true maximum. Therefore, we can deduce that $h^2_{max} > H^2$ simply by evaluating the derivative at H^2.

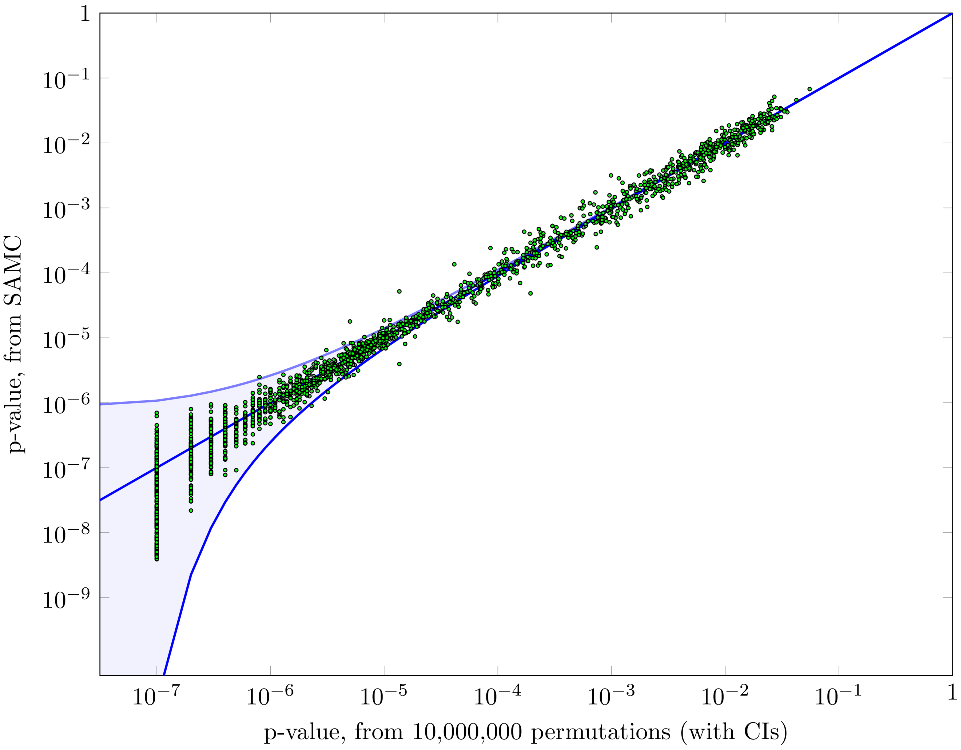

Supplementary Figure 7 Performance of SAMC. p-values from 10,000,000 permutations, compared to SAMC p-values with t_0=1,000 and 1,000,000 permutations (in log scale). Evaluated on 1,907 methylation sites, as in Supplementary Figure 2, with 99.95% CIs. SAMC is reasonably calibrated, also for small p-values.

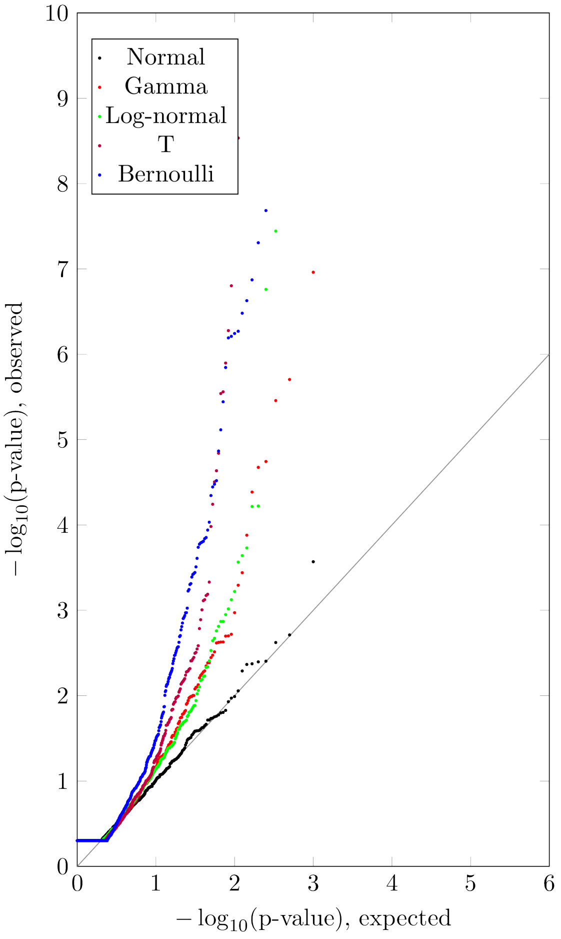

Supplementary Figure 8 Sensitivity to outliers. The QQ-plots of parametric p-values are shown for phenotypes generated from the assumed normal distribution, as well as from several heavy-tailed distributions. When deviating from normality, skewness in p-values is observed.

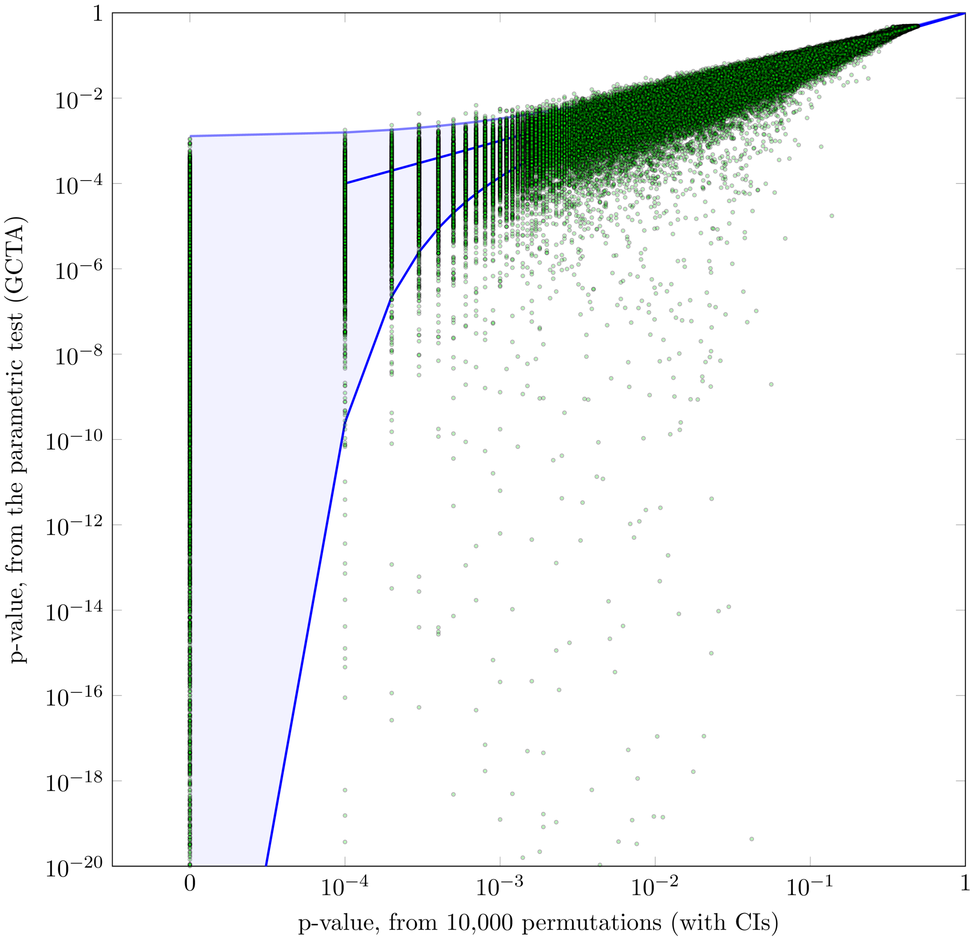

Supplementary Figure 9 Discrepancy in p-values, with outliers removed. p-values from 10,000 permutations, compared to GCTA p-values assuming asymptotics (in log scale). Removal of outliers does not eliminate discrepancies.

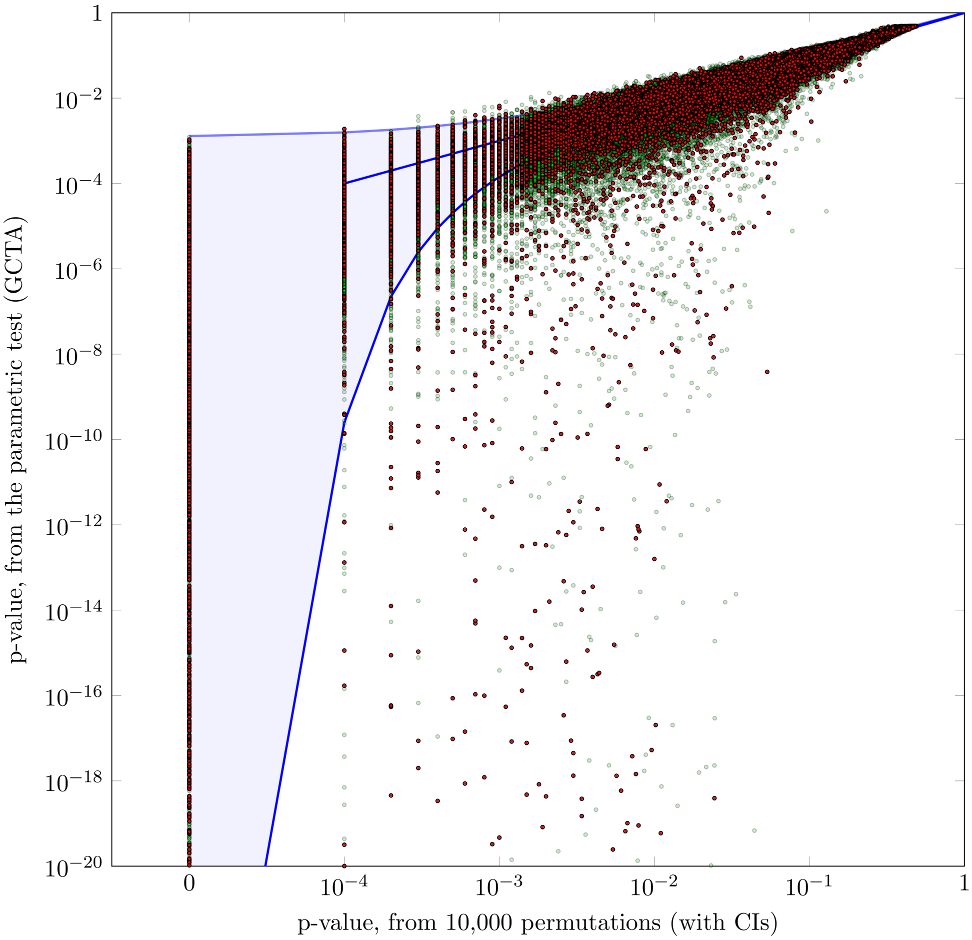

Supplementary Figure 10 Sites with SNPs in probes. The KORA analysis is shown here, with sites with known SNPs in their probes colored in red. Such sites are likely to be multimodal. Removal of such sites does not discard all discrepancies.

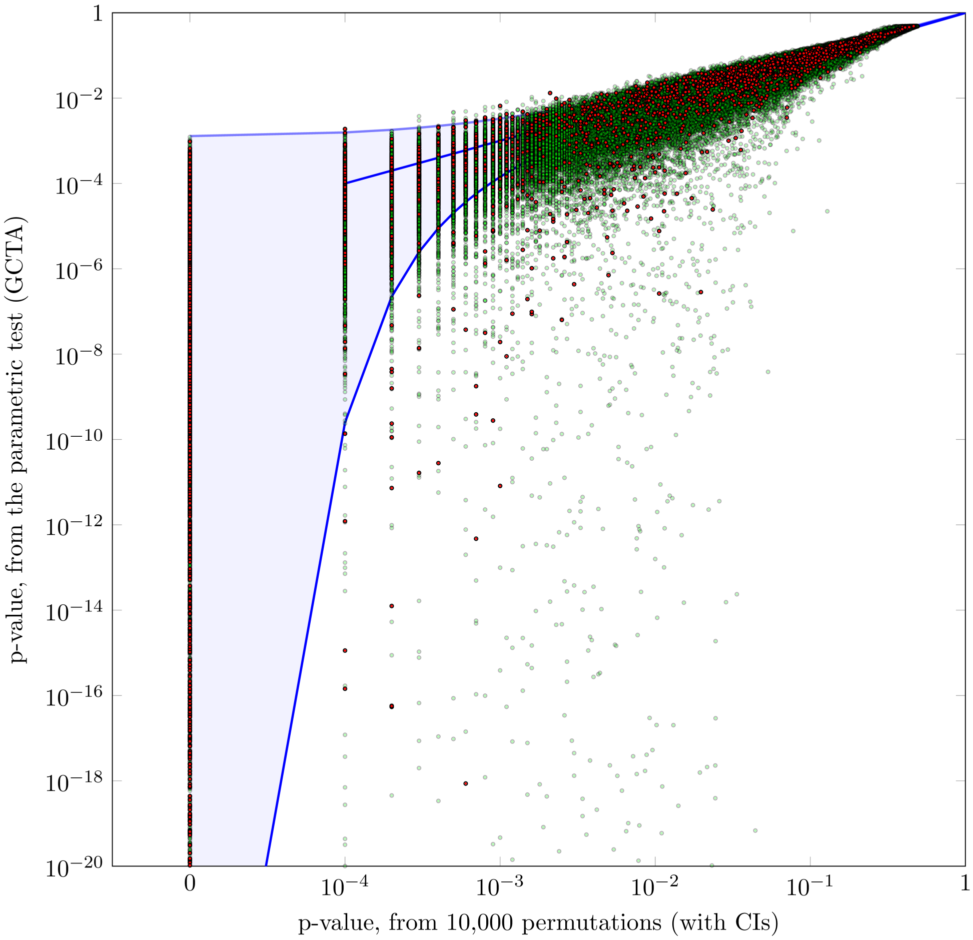

Supplementary Figure 11 Sites with a multimodal distribution. The KORA analysis is shown here, with sites with a multimodal distribution, as detected by *gaphunter*, colored in red. Removal of such sites does not discard all discrepancies.

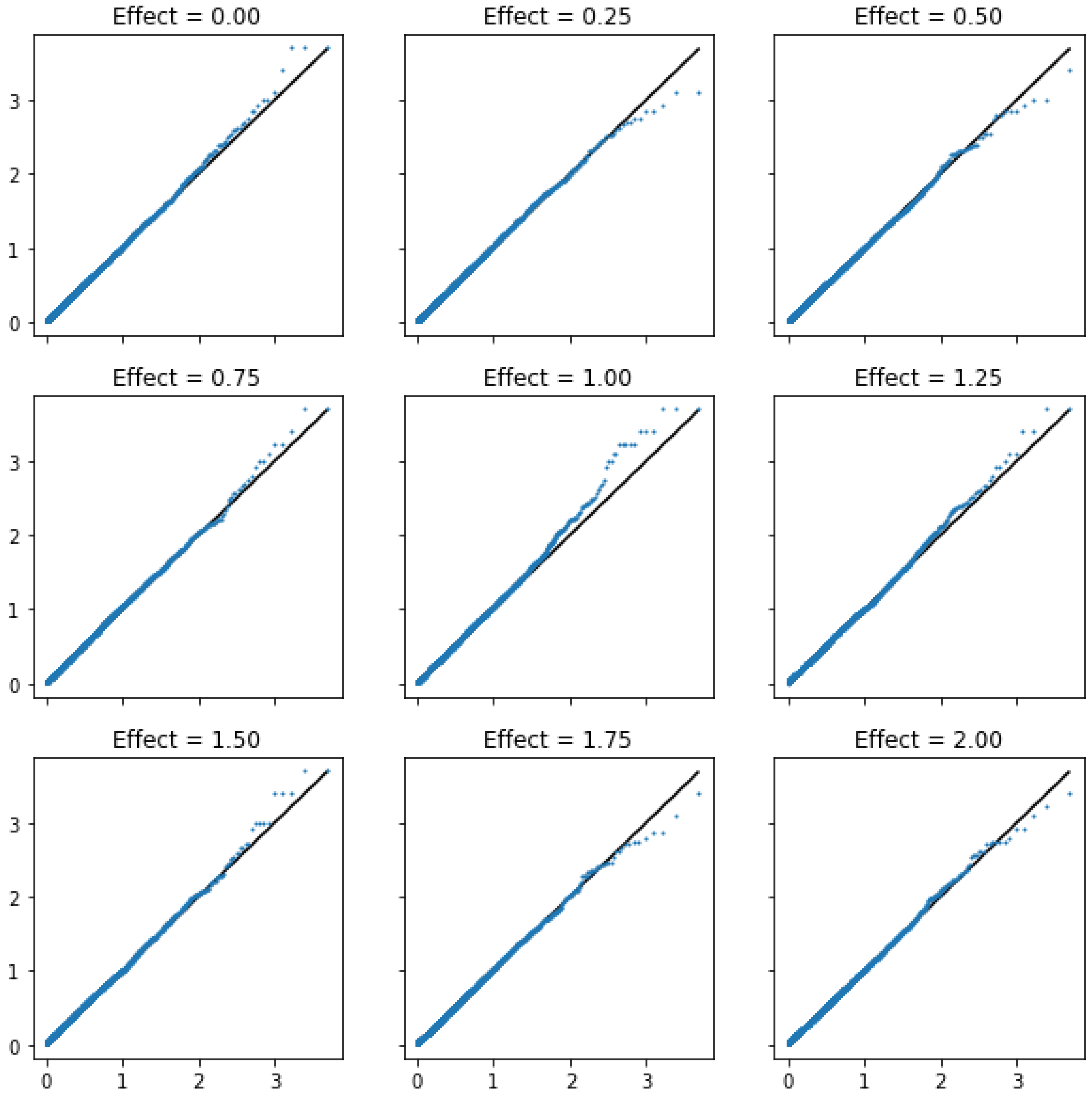

Supplementary Figure 12 QQ-plots for permutation p-values with covariates. Permutation p-values were calculated for phenotypes simulated from the KORA dataset with varying effect sizes. See text for details.

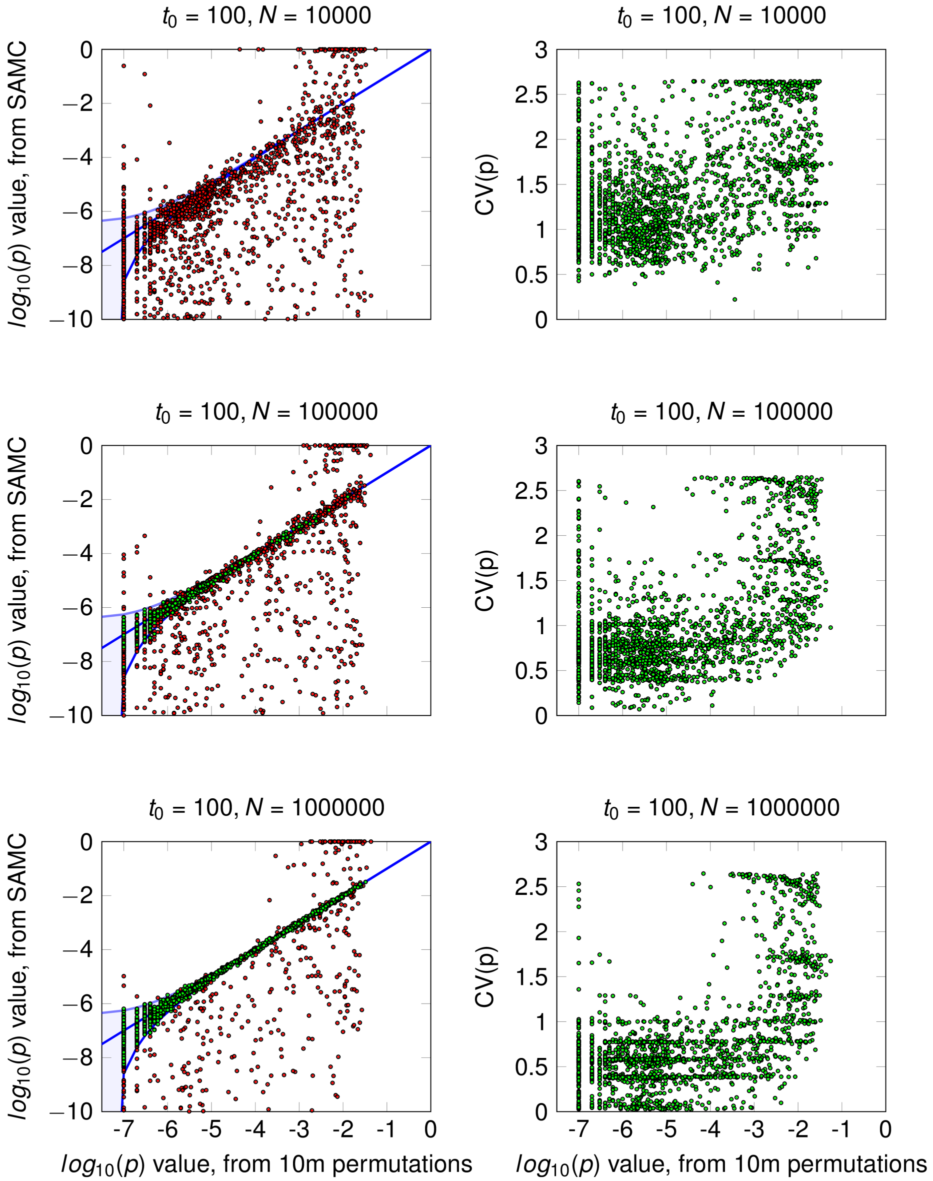

Supplementary Figure 13 Performance of SAMC - various parameters. p-values from 10,000,000 permutations, compared to SAMC p-values with various t_0 and N (in log scale). Evaluated on 1,907 methylation sites, as in Supplementary Figure 2. Red dots depict sites with RSE $> 10\%$. Data shown for $t_0 = 100$ as the number of permutation N grows (left panes). The coefficient of variation (sample standard deviation divided by sample mean) across 8 runs for the same set of parameters (right panes).

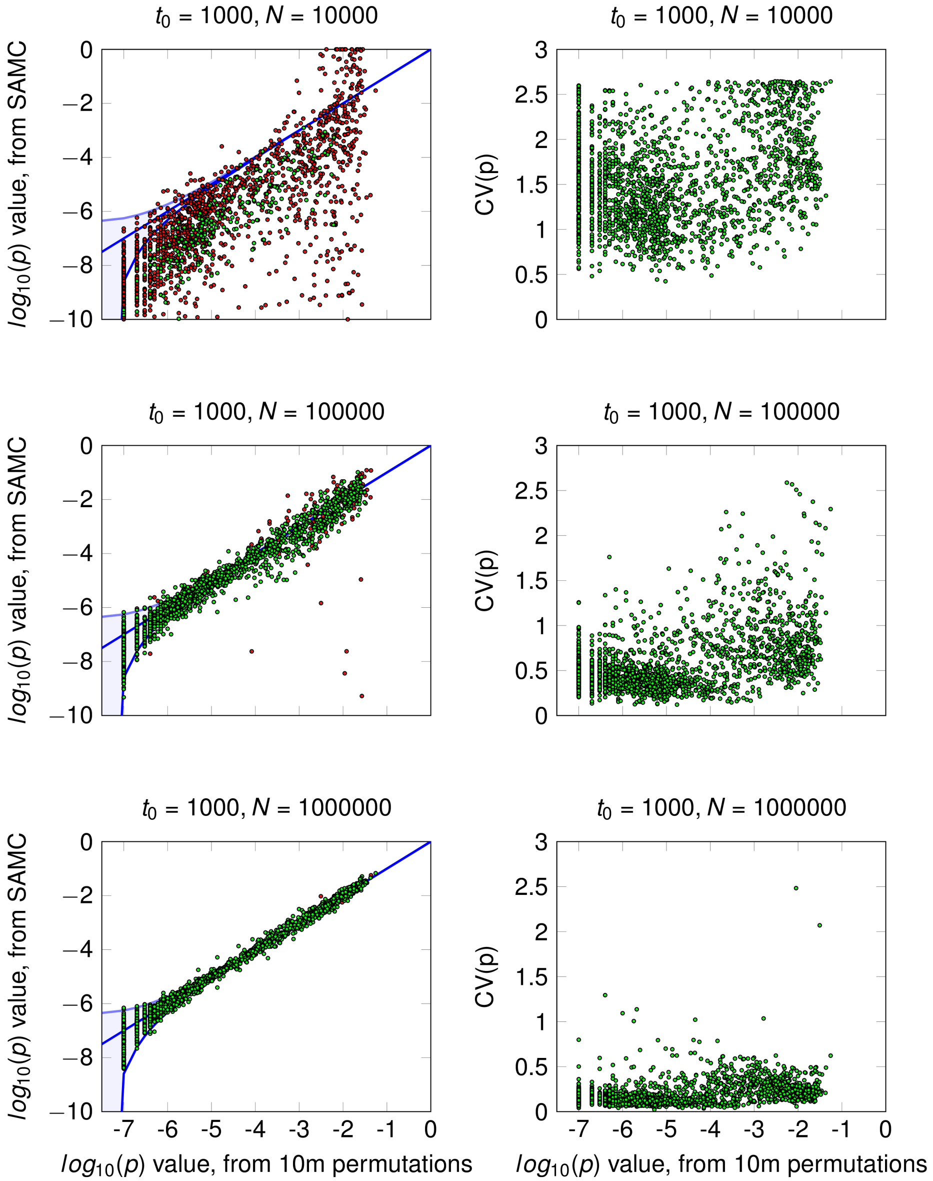

Supplementary Figure 14 Performance of SAMC - various parameters. Performance and CV for $t_0 = 1000$ (see Supplementary Figure 13 for details).

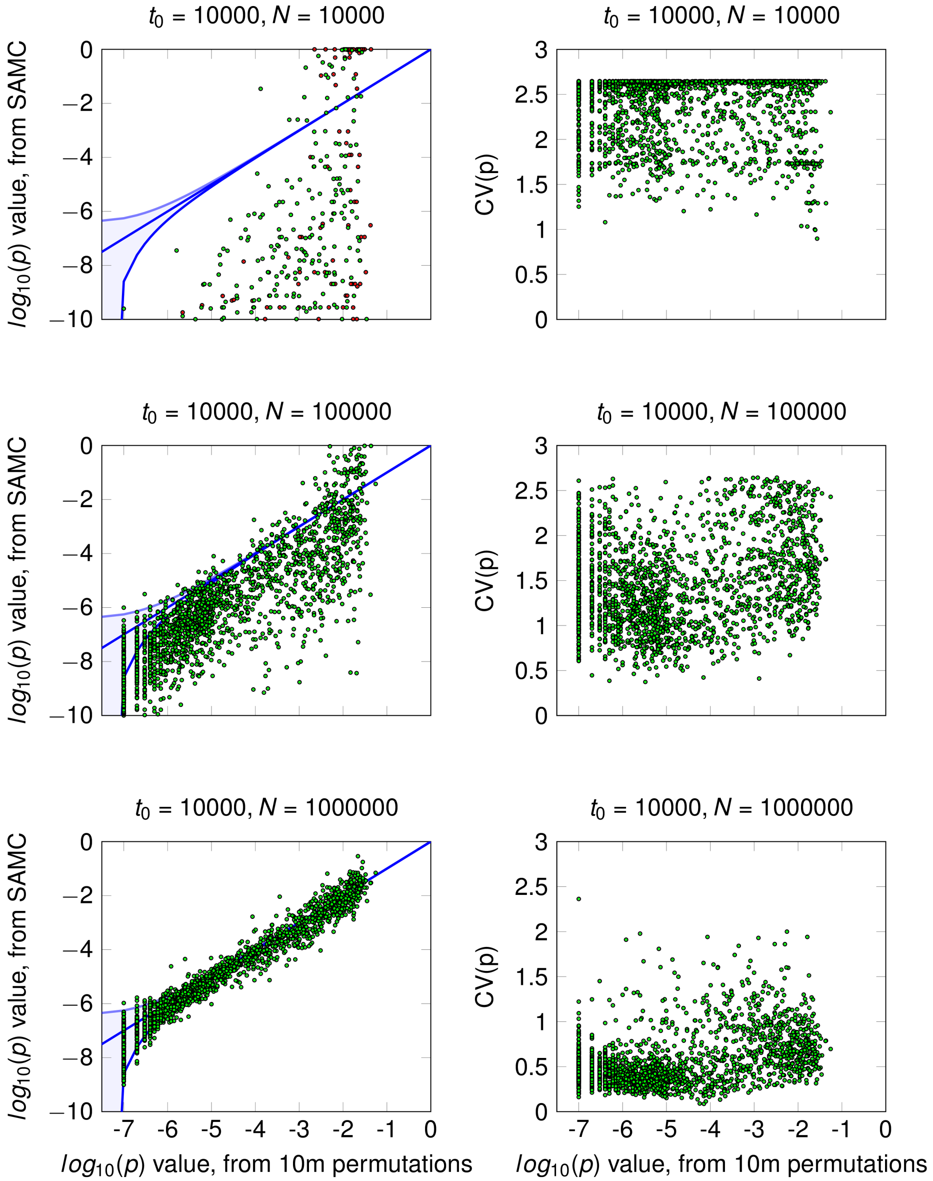

Supplementary Figure 15 Performance of SAMC - various parameters. Performance and CV for $t_0 = 10000$ (see Supplementary Figure 13 for details).

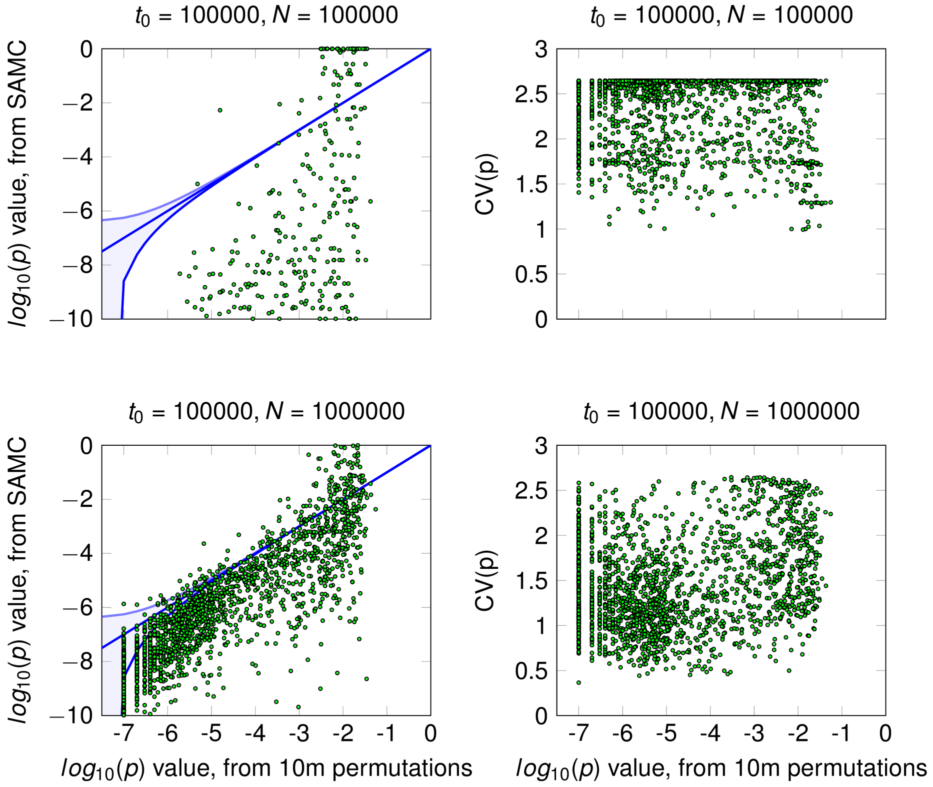

Supplementary Figure 16 Performance of SAMC - various parameters. Performance and CV for $t_0 = 100000$ (see Supplementary Figure 13 for details).

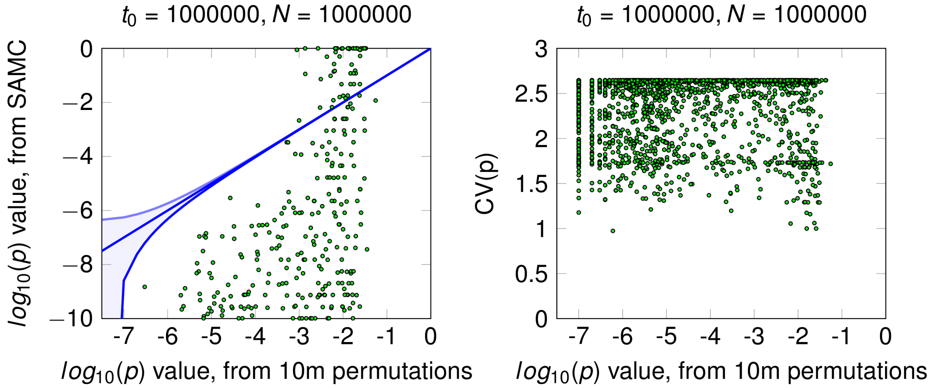

Supplementary Figure 17 Performance of SAMC - various parameters. Performance and CV for $t_0 = 1000000$ (see Supplementary Figure 13 for details).

Supplementary Tables

Site	Parametric p-value	Param. p-value (QN)	Perm. p-value	CI
cg00123214	$2.601 \cdot 10^{-08}$	$1.468 \cdot 10^{-05}$	$12 \cdot 10^{-09}$	$(6.201, 20.962) \cdot 10^{-09}$
cg00044796	$3.779 \cdot 10^{-13}$	$2.656 \cdot 10^{-11}$	$4 \cdot 10^{-09}$	$(1.090, 10.242) \cdot 10^{-09}$
cg01821635	$3.941 \cdot 10^{-18}$	$1.209 \cdot 10^{-15}$	$2 \cdot 10^{-09}$	$(0.242, 7.225) \cdot 10^{-09}$
cg06784218	$5.756 \cdot 10^{-21}$	$1.045 \cdot 10^{-19}$	$4 \cdot 10^{-09}$	$(1.090, 10.242) \cdot 10^{-09}$
cg08963013	$4.625 \cdot 10^{-26}$	$9.038 \cdot 10^{-21}$	$6 \cdot 10^{-09}$	$(2.202, 13.059) \cdot 10^{-09}$
cg14252149	$5.005 \cdot 10^{-34}$	$6.202 \cdot 10^{-26}$	$4 \cdot 10^{-09}$	$(1.090, 10.242) \cdot 10^{-09}$
cg00071950	$5.678 \cdot 10^{-36}$	$2.553 \cdot 10^{-35}$	$5 \cdot 10^{-09}$	$(1.623, 11.668) \cdot 10^{-09}$
cg02002194	$9.238 \cdot 10^{-42}$	$1.812 \cdot 10^{-43}$	$4 \cdot 10^{-09}$	$(1.090, 10.242) \cdot 10^{-09}$
cg11266682	$1.873 \cdot 10^{-46}$	$8.708 \cdot 10^{-44}$	$7 \cdot 10^{-09}$	$(2.814, 14.423) \cdot 10^{-09}$
cg15296535	$8.427 \cdot 10^{-51}$	$5.565 \cdot 10^{-53}$	$9 \cdot 10^{-09}$	$(4.115, 17.085) \cdot 10^{-09}$

Table 1 Effect of quantile normalization on extreme p-values. For 10 selected sites, we show the analyical parametric p-value, as calculated by GCTA; the parametric p-value calculated on the phenotypes after quantile normalization (QN); the permutation p-value, as calculated by 10^9 permutations, and the CIs implied by it. QN does not eliminate the discrepancy between the parametric and the permutation test.

Supplementary Notes

Supplementary Note 1 Reasons for p-value discrepancy

We discuss the underlying reasons for p-value discrepancies.

Power differences. It is possible that the permutation test is underpowered relative to the parametric test. To this end, we conducted a simulation study. We simulated phenotypes with varying degrees of heritability under the LMM (500 phenotypes per h^2 value), and used both tests to calculate p-values (using N=10,000 permutations for the permutation test). We calculated their false positive rate (FPR) and their power as a function of the true h^2 value (Supplementary Figure 4), for thresholds of $p = 0.1, 0.01$ and 0.001. The FPR is approximately well-calibrated (10.3%, 1.3% and 0.4%, respectively, for the parameteric test; 10.6%, 1.4%, 0.3% for the permutation test), while the power of the two tests is comparable, in line with existing literature [1, 2]. We conclude that the permutation test is equally powerful under the LMM, and thus that reduced power does not explain the discrepancy.

Normalization. A common practice for heritability estimation is to perform some type of normalization on the phenotypes before analysis, such as quantile normalization (QN), which ensures that the phenotype follows an empirical normal distribution. Performing QN can often increase proper p-value behavior. However, QN may also reduce power, e.g. when the phenotype is bimodal, QN will unify the two modes into a single unimodal distribution, and will the discard the important information of the mode to which each sample belongs. Additionally, we note that QN should be applied to the residuals and not to the phenotype itself, which is problematic in the presence of strong unknown covariates. To this end, we applied QN to our data, and calculated parametric p-value vs. the permutation p-values, as in Supplementary Figure 1. The results, shown in Figure 3, indicate that while QN indeed reduces the number of inflated (small) p-values, many sites remain with substantial discrepancy. Also, as expected, QN introduced many deflated p-values, i.e., sites whose parametric p-values are much larger than their permutation p-value counterpart. We additionally reran the analysis of Table 3, comparing permutation p-values with parameteric p-values, with and without QN, on 10 sites with small permutation p-values (Table 1). Again, QN fails to eliminate the large discrepancies. We conclude that while QN may be useful in many cases, it does not alleviate the discrepancies at hand.

Wrong asymptotic distribution for statistic under the model. Another potential source for such discrepancies is the distribution assumed for the statistic used for the cal-

culation of the p-value, even when the null model is correct. The assumptions underlying p-value calculation are often violated, leading to inaccurate p-values. However, our simulation study above shows the FPR is well calibrated, so the asymptotic distribution is expected to be a reasonable approximation. As a method for the correction of the distribution of p-values for mild deviations from the asymptotic distribution, it was suggested [3] to consider a larger parametric family of distributions for the distribution of the LR statistic: $\Pr(2\log\Lambda = x) = w \cdot \chi^2_0(x) + (1-w)a \cdot \chi^2_d(x)$, where Λ is the LR statistic, and w, d and a are constants to be fitted. A small number of permutations (e.g., 1,000) is used to generate a sample of LR statistics, and they are used to find the best fitting model of a weighted mixture of a constant zero and a chi-square distribution, with an arbitrary number of degrees of freedom. We followed the suggested procedure and obtained adjusted p-values. While it was possible to slightly adjust the bias, the results are qualitatively the same, with large discrepancies remaining unchanged (data not shown). We conclude that this is not the source of discrepancy.

We finally considered model mis-specification, where the model is improper for the data at hand, and in particular, the assumption that the phenotype takes a multivariate normal distribution.

Sensitivity to outliers. In line with existing literature [4], we observed large p-value deviations in phenotypes simulated to have heavy-tailed marginal distributions, which cause relatively distant outliers. Specifically, we generated phenotypes by drawing phenotype measurements i.i.d. from a target distribution. When the target distribution is a normal distribution, this amounts to the model null hypothesis. For any target distribution, the permutation test is assured to be well-calibrated due to the exchangeable distribution of the phenotype. However, the parametric p-values might not be calibrated. To this end, we generated 1,000 phenotypes, from several heavy-tailed target distributions, calculated their parametric p-values using the KORA kinship matrix, and constructed a quantile-quantile (QQ) plot to examine their calibration.

The results are shown in Supplementary Figure 8. For a normal distribution, the QQ-plot is aligned, with the smallest p-value reaching about 10^{-3}, as expected, while, for other distributions, skewness is observed. In particular, we show the QQ-plots for the Gamma distribution with parameters $\alpha = 1/2, \beta = 1$, the standard log-normal distribution, the T distribution with 2 degrees of freedom, and the Bernoulli distribution with $p = 0.99$.

We further tested the effect of outliers on the data at hand. The GTEx dataset, did not contain any outliers, while the KORA dataset did contain 2-5 outliers, as visible in a PCA plot (not shown). We removed all 5 outliers any re-ran a comparison between permutation and parametric p-values and verified that many large discrepancies remain (Supplementary Figure 9). Therefore, removal of outliers does not solve the problem.

Empirical discrepancies extend beyond multimodality. We have seen that multimodality (e.g. a binary Bernoulli distribution) may be the cause of discrepancy. Indeed, analyzing the KORA dataset, we have seen that many of the problematic sites exhibit a bimodal or trimodal behaviour. Thus, we set out to find if such sites are the ones displaying discrepancy.

First, we removed sites with known multimodality. In particular, having a SNP in the probe of a site causes the hybridization of the probe to depend on the genotype in some cases, which may cause multimodal phenotypes. We removed 57,341 such sites, obtained from [5] from our analysis; however, the remaining sites still exhibit large p-value discrepancies (Supplementary Figure 10), qualitatively similar to Supplementary Figure 1.

Second, we explicitly removed sites empirically seen to be multimodal. Current quality-control (QC) methylation pipelines typically remove potentially problematic probes as those with DNA methylation distributions characterized by two or more distinct clusters separated by gaps. We used the *gaphunter* program [6] with default parameters to identify and remove 3270 multimodal sites. However, remaining sites still show discrepancies, as seen in Supplementary Figure 11. Further examination shows that those are indeed sites with one mode but several scattered outliers. We note that as these outliers may be different within each site, it is not possible to only remove a small amount of samples to avoid outliers. Indeed, using *gaphunter* to remove these sites as well (using a flag which includes multimodal sites with a very small cluster size) removed 193,762 of 431,366 total sites (45%), well beyond any reasonable QC.

Supplementary Note 2 Validation of likelihood function behaviour

The following was tested on each one of the 7,989 methylation site phenotypes on chromosome 22 of the KORA dataset, and on 10,000 random permutations of each site, resulting in a total of 79,897,989 $\approx 10^{7.9}$ phenotypes.

First, we evaluated the sign of the derivative of the log-restricted-likelihood function at $h^2 = 0, 0.1, \ldots, 0.9, 1$, as described in the Methods section. We checked whether there was indication of more than one local maximum of the log-restricted-likelihood function, by testing if more than one of the follow cases hold: (i) the derivative at $h^2 = 0$ is negative (indicating a local maximum at 0); (ii) the derivative at $h^2 = 1$ is positive (indicating a local maximum at 1); (iii) there are two adjacent grid points a and b such that the derivative is positive at $h^2 = a$ and negative at $h^2 = b$ (indicating a local maximum between them). For all phenotypes tested, there was no indication of more than one local maximum.

Second, we estimated the heritability of each phenotype (using REML, as described in

Methods), and verified that the sign of the derivative at the grid points was consistent with the estimate, where (i) if the estimate is 0, we expect the derivative at 0 to be negative; (ii) if the estimate is 1, we expect the derivative at 1 to be positive; (iii) at all other grid points, we expect the derivative to be positive if and only if the estimate is larger than the grid point. Again, for all phenotypes tested, all the derivative signs were consistent with the estimate.

Supplementary Note 3 Using the permutation test for LMM with covariates

We discuss the application of permutation testing in LMM when covariates are involved.

1 Potential issues

In order for the permutation test to be calibrated under the null hypothesis, the distribution of the permuted quantity needs to be exchangeable, i.e., having the same distribution when its entries are exchanged. In particular, any multivariate distribution with i.i.d. entries is exchangeable. Without covariates, the LMM null hypothesis is $\mathbf{y} \sim \mathcal{N}(\mathbf{0}_n, \sigma_p^2 \mathbf{I}_n)$. With the constant intercept covariate, it is $\mathbf{y} \sim \mathcal{N}(\beta \cdot \mathbf{1}_n, \sigma_p^2 \mathbf{I}_n)$. In both those cases, the distribution is exchangeable, resulting in an exact permutation test.

When additional, non-constant, covariates are added, the exchangeable quantity is the vector of residuals, $\mathbf{e} = \mathbf{y} - \mathbf{X}\boldsymbol{\beta}$. However, it is generally not observed, and thus needs to be estimated from the data. This is a limitation compared to the permutation test without non-constant covariates, due to mainly two reasons.

The first reason is that our test is no longer completely non-parametric, as we now explicitly assume a model used for estimating the residuals given the phenotype and the covariates. This is still an improvement over a completely parametric test, as we still do not assume an explicit form for the distribution of the residuals, which allows for flexible modeling; see also the discussion above regarding non-normal i.i.d. errors. This is in line with other non-parametric approaches in genetics [7].

The second issue is that even if the residual model is correct, our estimate may not be precise due to the need to estimate the unobserved parameters. In the linear example above, we have no access to the true $\boldsymbol{\beta}$, and thus we need to estimate $\hat{\boldsymbol{\beta}}$ from $\mathbf{y}$ and $\mathbf{X}$. If, for example, the estimate $\hat{\boldsymbol{\beta}}$ was far from its true value $\boldsymbol{\beta}$, $\hat{\mathbf{e}}$ will have a component of $\mathbf{X}$ that did not exist in $\mathbf{e}$ (as $\hat{\mathbf{e}} - \mathbf{e} = \mathbf{X}(\hat{\boldsymbol{\beta}} - \boldsymbol{\beta})$). If, in addition, $\mathbf{X}$ has a covariance structure similar to $\mathbf{K}$ (equivalently, $\mathbf{X}$ are correlated with the dominant eigenvectors of $\mathbf{K}$), this could induce a false correlation that did not originally exist, inflating the p-value.

See [8–10] for a detailed discussion in the similar simpler case of linear regression, whose conclusions carry to this case. Luckily, with even small sample sizes, we can expect the bias in estimating $\boldsymbol{\beta}$ to be small [10].

2 Dealing with covariates

With these potential issues in mind, our method supports two approaches to dealing with covariates.

2.1 Block permutation for discrete covariates

Consider the case where there are few discrete covariates (e.g. sex and smoking status). Covariates partition the samples into a small (e.g. 4) number of groups, according to their covariate values (e.g. non-smoking males). In this case, we can avoid any potential issues with confounding by covariates by conditioning on them. This is done in the simple manner of permuting individuals only within each group. Our software package thus supports drawing random permutations only from the set of permutations maintaining the group block structure. Our method remains unchanged otherwise.

2.2 General covariates

In the case of general covariates, we apply our method, extended to covariates. First, the likelihood derivative can be calculated in linear time also with covariates [11]. The covariates are permuted along with the phenotype, i.e. per permutation π, we compare $\pi(\mathbf{y}), \pi(\mathbf{X})$ vs. the unchanged $\mathbf{K}$. Note that the implicit underlying residual model here, based on REML, is the linear $\mathbf{e} = \mathbf{y} - \mathbf{X}\boldsymbol{\beta}$.

It is important to note a distinction between testing for heritability and the commonplace scenario of testing for an association between a phenotype and a single marker. In the former, the tested quantity is h^2, while in the latter, one of the covariates is the marker of interest, and the tested quantity is its associated fixed effect. A permutation test is often applied in the single-marker scenario, and its properties and pitfalls are discussed elsewhere, e.g., [12]. While several issues are common, e.g. inflation in the presence of confounders, the heritability testing scenario is in a sense simpler - there are only three distinct entities: the phenotype $\mathbf{y}$, the covariates $\mathbf{X}$ and the kinship matrix $\mathbf{K}$. In the single-marker scenario, there is an additional entity, the tested marker, and its additional relationships to the others adds complications.

3 Empirical study

To validate the permutation test with covariates, we performed an empirical study. First, we performed a simulation study. Using the KORA kinship matrix and the age, sex, and smoking status covariates (see Methods), we simulated phenotypes under the null hypothesis of no heritability, as $\mathbf{y} \sim \mathcal{N}(\mathbf{X1} \cdot \beta, \mathbf{I}_n)$, with varying effect sizes $\beta = 0, 0.25, \ldots, 2$. For each effect size, we generated 100 random phenotypes, and performed a permutation test with 10,000 permutations. We used a quantile-quantile plot (QQ-plot) to verify that the p-values are well calibrated (Supplementary Figure 12). The QQ-plots were calibrated for all effect sizes, showing that the bias in estimating $\boldsymbol{\beta}$ [10] is indeed negligible. A similar simulation study with GTEx gave qualitatively similar results (not shown).

In addition, as discussed in the main text, we compared parametric and permutation p-values for the KORA dataset, both with and without covariates. While the p-values for the same phenotype obviously differ, under both regimes there are significant discrepancies, showing that they are not due to the lack (or addition) of covariates.

4 Summary

While permutation testing is mathematically exact only with constant covariates and an exchangeable distribution, we and others have shown, in theoretical analyses, data studies and simulations, that under a wide range of scenarios, permutation testing remain calibrated with covariates as well.

In practice, there are endless combinations of error distributions, correlations between samples, covariates, markers and phenotypes, and more. A complete mapping of the robustness of both the parametric and permutation tests remains a subject for a future study [4], and is beyond of the scope of this work. We note that despite these potential issues, in many cases the parametric alternatives suffer from significantly larger practical problems. An informed choice between the two alternatives, comparing their advantage and drawbacks, remains the role of the researcher.

Supplementary Note 4 The SAMC algorithm

1 Algorithm description

The Stochastic Approximation Markov Chain Monte Carlo (SAMC) algorithm can be generally described as an iterative, stochastic approximation method, that, given a partitioning of the sample space into subsets, can estimate the subset sizes. SAMC can be used for efficient p-value calculation [13]. For the sake of completeness, we give here a presentation

of the SAMC algorithm in the context of p-values; for a full description and explanation, see [13, 14].

Given an estimate H^2, we want to calculate its p-value, i.e. the probability of the event $\hat{h}^2(\pi(\mathbf{y})) \geq H^2$. We define a partitioning of $[0, 1]$ to $D + 1$ intervals, where the interval $[0, H^2]$ is divided into D equally sized intervals, and $[H^2, 1]$ is an additional interval. This induces a partitioning of the permutation space - if $[h_d^2, h_{d+1}^2]$ is the d-th interval, then we define $\mathcal{X}_d = \{\pi | \hat{h}^2(\pi(\mathbf{y})) \in [h_d^2, h_{d+1}^2]\}$; namely, the set of permutations of the phenotype for which the estimated heritability value falls in the interval $[h_d^2, h_{d+1}^2]$. Then, $\mathcal{X}_1 \cup \ldots, \cup \mathcal{X}_{D+1}$ is a full partitioning of the space of permutations. Then, the p-value is exactly the probability of the event $\mathcal{X}_{D+1}$ assuming a uniform distribution over permutation space, or $|\mathcal{X}_{D+1}|/n!$.

SAMC utilizes the following key observation. Let $p_i = |\mathcal{X}_i|/n!$ be the true sizes of the subsets, and let $\hat{p}_i$ be some estimate of their sizes. Let $\psi(i) = (1/\hat{p}_i)/(\sum_{i=1}^{D+1}(1/\hat{p}_i))$, that is, a probability distribution on subsets, proportional to $1/\hat{p}_i$. This probability distribution is constructed to have the self-balancing property introduced below. For a permutation $\pi \in S_n$, let $J(\pi)$ be the index of the interval in which $\hat{h}^2(\pi(\mathbf{y}))$ falls. We are interested in the probability distribution over permutations, defined as:

$$f(\pi) \propto \psi(J(\pi))$$

The key property of f is that if the estimates $\hat{p}_i$ are accurate, then sampling from f will sample uniformly from the subsets. To see that, for a subset $\mathcal{X}_i$

$$\Pr_{\pi \sim f}(\pi \in \mathcal{X}_i) = \sum_{\pi \in \mathcal{X}_i} f(\pi) \propto \frac{|\mathcal{X}_i|}{n!} \cdot \psi(i) \propto p_i/\hat{p}_i.$$

If $\hat{p}_i = p_i$, then $\Pr_{\pi \sim f}(\pi \in \mathcal{X}_i) \propto 1$, and thus the subset sampling is uniform. In order to sample from f, a Metropolis-Hastings (MH) step is used. The target distribution for the MH step is f, which in turn is constructed from the current estimates $\hat{p}_i$. The choice of a well performing proposal distribution q is discussed below.

The update rule for subset probability estimates follows the stochastic approximation (SA) algorithm [15], which ensures that the estimates can be improved continuously as the simulation goes on. The SA update rule defines a sequence of weights, called gain factors, designed to ensure convergence.

Therefore, the algorithm alternatingly samples a new random permutation according to the target distribution f defined by current estimates $\hat{p}_i$, using the MH sampling algorithm; and then, given the subset in which the new permutation falls, updates the estimates. Informally, given the above partitioning, SAMC will eventually sample from each subset uniformly, while in the process estimating the probability of each subset. When the algo-

rithm converges, its estimate for the last subset will be our estimate of the p-value.

In practice, as in [13], we will update an estimate of the log of a value only proportional to the probability. That is, defining $\theta_i^{(t)} = \log \hat{p}_i^{(t)}$ to be our updated estimate at iteration t, then our estimated probability for subset i is:

$$\hat{p}_i = \frac{\exp(\theta_i^{(t)})}{\sum_{d=1}^{D+1} \exp(\theta_d^{(t)})}.$$

The proposal distribution $q(\pi_t, \tau)$ defines the probability of choosing a new permutation τ, given that the current permutation is π_t. Let $\mathbf{e}_i = (0, \ldots, 0, \underbrace{1}_{i}, 0, \ldots, 0)$, Finally, the algorithm is:

1. Initialize a uniform estimate, $\theta_1^{(t)} = \ldots, = \theta_{D+1}^{(t)} = 0$.
2. Choose a random initial permutation π_1.
3. For $t = 1, \ldots, T$ (or until convergence):
    (a) Simulate a sample π_{t+1} by a single Metropolis-Hastings update, as follows:
        i. Generate τ according to the proposal distribution $q(\pi_t, \tau)$.
        ii. Calculate the ratio $r = \exp(\theta_{J(\pi_t)}^{(t)} - \theta_{J(\tau)}^{(t)}) \cdot q(\tau, \pi_t)/q(\pi_t, \tau)$
        iii. Accept the proposed move with a probability of $min(1, r)$. If accepted, set $\pi_{t+1} = \tau$. Otherwise, set $\pi_{t+1} = \pi_t$.
    (b) Update the estimates: For $i = 1, \ldots, D+1$, set $\theta_i^{(t+1)} = \theta_i^{(t)} + \gamma^{(t)}(\mathbf{e}_{J(\pi_{t+1})} - (\frac{1}{D+1}, \ldots, \frac{1}{D+1}))$, where $\gamma^{(t)}$ is called the *gain factor* and is defined as $\gamma^{(t)} = t_0 / \max(t_0, t)$.
4. Return $\exp(\theta_{D+1}^{(t)}) / \sum_{i=1}^{D+1} \exp(\theta_i^{(t)})$.

Note that this algorithm does not require us to fully estimate the heritability of a permutation τ, but rather, only to calculate in which interval $J(\tau)$ it falls. For each interval $[h_d^2, h_{d+1}^2]$, we can check the derivatives at the endpoints h_d^2 and h_{d+1}^2. If the derivative is positive at the h_d^2 and negative at h_{d+1}^2, then we know a maximum exists within that interval. Using the derivative allows us to avoid the heritability estimation step, as before.

An additional speedup is obtained using the following. The proposal distribution altered only a small proportion of the permutations between consecutive steps (see below). Therefore, the quantity $\mathbf{u}_{t+1} = \mathbf{U}^T \pi_{t+1}(y)$ may be calculated from $\mathbf{u}_t = \mathbf{U}^T \pi_t(y)$ by adding a small number of columns of $\mathbf{U}^T$.

Tuning the parameters of SAMC

We now describe guidelines for tuning the parameters of SAMC algorithm described above. These are the proposal distribution q, the number of intervals, D; the number of iterations, N; and an additional parameter, t_0, that corresponds to the number of iterations after which the estimation will begin converging more rapidly. In more detail, the estimate per subset is updated in the t-th iteration by a value which is multiplied by a weight γ_t, the gain factor, which effectively controls the magnitude of the update. The gain factor is 1 for the first t_0 iterations, and then it begins decreasing by defining $\gamma_t = t_0/t$ for $t > t_0$.

Proposal distribution. We follow [13] in defining $q(\pi_t, \tau)$ as the uniform distribution over all permutations that are generated by randomly permuting 5% of the values of π_t. Results with 10% were qualitatively similar (not shown).

Number of intervals. SAMC in general prefers a fine partition, so that the transition between intervals will be smooth. On the other hand, a large number of subsets will require a larger number of iterations (permutations) until each subset has been visited. We found that $D = 50$ or $D = 100$ are often a reasonable trade-off; a D as low as $D = 20$ may be suitable as well. We refer the reader to [13] for a further discussion about partitioning.

Number of iterations and t_0. It is important that t_0 will not be too small; otherwise, estimates will begin to converge before all subsets have been sufficiently visited. Previous recommendations have advised t_0 =1,000 to 5,000 or $t_0 = 2D$ to $100D$. On the other hand, γ_t should be very close to 0 at the end of simulations. Otherwise, the resulting estimates will have a large variation. This can be controlled by determining the ratio between T and t_0, which controls the last gain factor as $\gamma_N = t_0/N$. Therefore, N should be sufficiently larger than t_0, e.g. $N = 100t_0$ or $1000t_0$. An alternative to setting a fixed N is to stop the algorithm when it seems to have sufficiently converged; we did not investigate this criterion in this paper.

Validation of parameter choice. In practice, the above rules of thumb are best validated empirically for the problem at hand. To this end, we recommend the following to check the performance of a given set of parameters. (i) First, run SAMC multiple (e.g. 5-10) times and compare the output p-value estimates. If there are large discrepancies, it might mean that convergence was not achieved. Further information can be gained by looking at the probability estimates for all subsets, not only the last. (ii) Second, as mentioned above, as SAMC converges, it visits all subsets approximately uniformly. The

relative sampling error (RSE) is defined as the maximal percentage of deviation of the sampling rate of any subset relative to the required uniform rate. For example, if $D = 50$ and the RSE is 10%, then each subset was visited by $1.9 \pm 0.19\%$ of permutations (there are 51 subsets and $1/51 \approx 0.019$). If the RSE is above some threshold, say 10%, then it is a strong indication that the run has not converged, and an increase of N is advised; (iii) Finally, if phenotypes exist whose heritability estimate has a relatively large p-value (e.g, 10^{-3}), run the simple (non-SAMC) permutation testing to get an estimate of the true permutation test p-value (e.g, with $N = 10^5$ or 10^6 random permutations), and compare the p-value estimated by SAMC. An additional possible approach we did not pursue is using the Potential Scale Reduction Factor [16], a convergence criterion based on a suitable comparison of the variance of sampled values within multiple runs, compared to the variance between them.

Parameter tuning for the KORA dataset. We chose parameters of SAMC by selecting several sites with a relatively high p-value, as estimated by 10,000 permutations. We examined the performance of SAMC with several parameter settings and compared it with the p-value obtained by the simple permutation test. We examined the effect of various parameter settings on the performance of SAMC, checking the relative sampling error (RSE) as well as the variability in estimates across several runs per site (see Methods, Supplementary Figure 13, Supplementary Figure 14, Supplementary Figure 15, Supplementary Figure 16 and Supplementary Figure 17).

We found, in agreement with the suggested parameter tuning guidelines of [13], that when t_0 is too small, convergence is slow - $t_0 = 1000$ appears to work well in our case. Moreover, when the ratio t_0/N is too large, estimates do not yet converge. That is, N must be sufficiently larger than t_0. We found $N \geq 100{,}000$ to give good results for $t_0 = 1000$. We concluded that selecting the right t_0 is important for a speedy convergence. We chose t_0=1,000 and N=1,000,000 as suitable parameters. Throughout the paper, we used $D = 50$ intervals.

Implementation. The permutation test was implemented in C++, using the Eigen [17] and Boost software libraries, and in Python.

Supplementary References

1. Fitzmaurice, G. M., Lipsitz, S. R. & Ibrahim, J. G. A note on permutation tests for variance components in multilevel generalized linear mixed models. *Biometrics* **63,** 942–946 (2007).
2. Schweiger, R. *et al.* RL-SKAT: an exact and efficient score test for heritability and set tests. *Genetics,* genetics–300395 (2017).
3. Listgarten, J. *et al.* A powerful and efficient set test for genetic markers that handles confounders. *Bioinformatics* **29,** 1526–1533 (2013).
4. Allison, D. B. *et al.* Testing the robustness of the likelihood-ratio test in a variance-component quantitative-trait loci–mapping procedure. *The American Journal of Human Genetics* **65,** 531–544 (1999).
5. Chen, Y.-a. *et al.* Discovery of cross-reactive probes and polymorphic CpGs in the Illumina Infinium HumanMethylation450 microarray. *Epigenetics* **8,** 203–209 (2013).
6. Andrews, S. V., Ladd-Acosta, C., Feinberg, A. P., Hansen, K. D. & Fallin, M. D. Gap hunting to characterize clustered probe signals in Illumina methylation array data. *Epigenetics & chromatin* **9,** 56 (2016).
7. Epstein, M. P. *et al.* A permutation procedure to correct for confounders in case-control studies, including tests of rare variation. *The American Journal of Human Genetics* **91,** 215–223 (2012).
8. Schmoyer, R. L. Permutation tests for correlation in regression errors. *Journal of the American Statistical Association* **89,** 1507–1516 (1994).
9. Anderson, M. J. & Robinson, J. Permutation tests for linear models. *Australian & New Zealand Journal of Statistics* **43,** 75–88 (2001).
10. Nyblom, J. in *Modern Nonparametric, Robust and Multivariate Methods* 69–90 (Springer, 2015).
11. Schweiger, R. *et al.* Fast and accurate construction of confidence intervals for heritability. *The American Journal of Human Genetics* **98,** 1181–1192 (2016).
12. Abney, M. Permutation testing in the presence of polygenic variation. *Genetic epidemiology* **39,** 249–258 (2015).
13. Yu, K., Liang, F., Ciampa, J. & Chatterjee, N. Efficient p-value evaluation for resampling-based tests. *Biostatistics* **12,** 582–593 (2011).
14. Liang, F., Liu, C. & Carroll, R. J. Stochastic approximation in Monte Carlo computation. *Journal of the American Statistical Association* **102,** 305–320 (2007).
15. Robbins, H. & Monro, S. A stochastic approximation method. *The annals of mathematical statistics,* 400–407 (1951).
16. Gelman, A. & Rubin, D. B. Inference from iterative simulation using multiple sequences. *Statistical science,* 457–472 (1992).
17. Guennebaud, G., Jacob, B., *et al.* *Eigen v3* http://eigen.tuxfamily.org. 2010.

18. Holle, R., Happich, M., Lwel, H., Wichmann, H., study group, M., *et al.* KORA-a research platform for population based health research. *Das Gesundheitswesen* **67,** 19–25 (2005).
